# Editorial: Perspectives on Carbon Dioxide Capture and Conversion

**DOI:** 10.3389/fchem.2021.664979

**Published:** 2021-04-14

**Authors:** Diana C. S. Azevedo, Sebastiao M. P. Lucena, Enrique Rodríguez-Castellón, Carlos Wexler

**Affiliations:** ^1^ Federal University of Ceara, Fortaleza, Brazil; ^2^ University of Malaga, Málaga, Spain; ^3^ University of Missouri, Columbia, KY, United States

**Keywords:** CO2, activated carbon, mesoporous silica, additive manufacturing, aluminophosphate molecular sieves, techno-economic analysis, ZIF–8

If we were to establish a year for the discovery of global warming caused by the concentration of greenhouse gases in the atmosphere, this year is 1988. In the summer of that year, the highest temperatures in the historical series of data collected so far were recorded. Moreover, atmospheric simulators had become so accurate that all existing models, validated from the reproduction of climate historical records, showed significant global warming when greenhouse gases were added (Weart, 2008). Today, more than 30 years later, the scientific community has produced a huge knowledge base around CO_2_ capture materials and processes, and the present research topic offers a nice sample of such efforts. From the point of view of materials, we present the prospects of conventional materials such as activated carbons, tailored to capture CO_2_ either as synthesized (Prauchner et al.) or by post-synthesis impregnation (Giraldo et al.). Mesoporous silicas belong to the group of very stable materials with a low CO_2_ adsorption capacity *per se*; however, they are preferential candidates for functionalization with amino silanes, which considerably increase their CO_2_ capture capacity (Vilarrasa-García et al.). The research topic also includes materials that weakly bind CO_2_ while keeping a relatively high uptake, such as the aluminum phosphate molecular sieves (Pérez-Botella et al.). Important techno-economic considerations of capture processes are considered (Danaci et al.), and the state of art of CO_2_ capture *via* mineralization of steel slag (Zhao et al.) is presented. Finally, new bed-forming techniques based on additive manufacturing are presented using the new material ZIF-8 (Verougstraete et al.) from the class of metal–organic frameworks (MOFs).

We hope that this collection will be useful both as an introduction to the subject of CO_2_ capture and as a relevant showcase of the latest research associated with the topic.
